# Learning styles of medical students from a university in China

**DOI:** 10.1186/s12909-023-04222-3

**Published:** 2023-04-12

**Authors:** Hai-ping Liu, Yue-hui Liu

**Affiliations:** 1 Haiping Liu College of Life Science and Food Engineering, Inner Mongolia Minzu University, Tongliao, 028043 Inner Mongolia China; 2 Affiliated Hospital of Inner Mongolia Minzu University, Tongliao, 028007 Inner Mongolia China

**Keywords:** Learning, Cognition, Surveys and questionnaires, Students, Medical

## Abstract

**Background:**

Investigating students’ learning styles can generate useful information that can improve curriculum design. This study adopts diverse measures to identify the learning styles of students despite limited literature related to clinical medical students in China. We utilized Felder’s Index of Learning Styles to examine the learning style characteristics of clinical medical students at Inner Mongolia Minzu University.

**Methods:**

Cluster sampling (probability sampling) was used. This cross-sectional study investigated clinical medicine students with regard to their learning style preference and the difference across genders. This study also analysed data collected from other published studies. A total of 411 students from the medical school at Inner Mongolia Minzu University completed the Index of Learning Styles Questionnaire. The questionnaire assessed the learning styles of students in four dimensions: visual-verbal learning, sequential-global learning, active-reflective leaning, and sensing-intuitive learning.

**Results:**

The analysis results show that clinical medicine students choose to receive visual information (73.97% of the student sample) instead of verbal information. These students prioritize sensory information (67.15%) rather than intuitive information and process reflective information (51.82%) rather than active information. They prefer to process information sequentially (59.85%) instead of globally. Our results also show that male students present a higher preference for an active learning style over a reflective learning style, while female students seem to present a higher preference for a reflective learning style over an active learning style. These preferences vary between cohorts (gender), but the difference is not statistically significant. Compared to data collected from other published studies, active, visual, sensing, and sequential are the most popular styles of learning adopted by medical science students.

**Conclusions:**

The identification of medical students’ learning style in China provides information that medical educators and others can use to make informed choices about modification, development and strengthening of medical educational programs. Our outcomes may potentially improve motivation, engagement and deep learning in medical education when used as a supplement to teaching/learning activities.

## Background

Many educators and psychologists believe that learning style (LS) is essential to students’ learning process. Applying learning style factors to teaching practice can improve learners’ learning efficiency [1]. Felder [1], Graf & Kinshuk [2], and others believe that when learners have apparent learning style preferences, matching teaching strategies with these preferred learning styles will facilitate the advantages of learning styles and improve students’ learning performance. In contrast, if teaching strategies do not match learners’ preferences, students will find it challenging to learn and master knowledge. Demirtas [3] and Mutlu [4] verified that a learning environment with an appropriate learning style enhances learners’ knowledge memory and application for course content and learning objects, respectively. According to Sangleto [5], research by Tseng [6], Graf [7], Popescu [8], and others shows that a learning style-based adaptive learning network environment has higher learning efficiency, can produce better user satisfaction, effectively reduces students’ learning time and improves their performance. As a result, verifying student learning styles, particularly educational and data processing priorities, can provide valuable information for constructing efficient learning actions.

Various inventories and evaluation metrics have been presented to evaluate individuals’ desired modes of data acquisition and processing or their preferred LS [9]. The Felder-Silverman model [10] is a popular model for measuring students’ learning styles in medical education. The metric established by the authors for measuring LS (Index of Learning Styles, ILS) employs the mentioned model [1]. The ILS is a straightforward metric constructed to evaluate students’ learning styles [1]. The ILS describes four domains, or fundamental perspectives, of LS priorities, each involving two opposite descriptors [11]. The ILS judges whether a student prefers to process data in a reflective or with an active approach using self-described priorities of obtaining data verbally or visually, determining data in universal or consecutive ways, and remembering and concentrating on sensorial data (what is heard and observed) or intuitional data (theories, opinions, and feasibilities). Some works have indicated that medical student (MSs) mainly prioritize a reflective learning style [12, 13]. Although various works have indicated that MSs also show an active learning style priority [14, 15], other studies have indicated no priority for active or reflective data processing [12]. Generally, works indicate that the popular learning styles among MSs are sensing, visual, and sequential [12–16]. However, opposite results were obtained by analysing gender deviations in MSs’ learning styles. Some empirical works have indicated the remarkable impact of gender on MSs’ learning styles [10, 12 and 17]. In contrast, certain studies have challenged the role of gender in the learning style preferences of students [13].

Little work has been devoted to using Felder-Silverman’s ILS to identify medical students’ learning style priorities in China. Thus, the current work attempts to answer the following questions:

1. What are the prevalent LSs among clinical medical students in China at the University of Inner Mongolia Minzu?
2. Are there considerable statistical deviations in students’ LSs due to their demographic features?
3. Are our clinical medical students’ learning styles compatible with those of other medical students?

## Methods

### The participants

The participants were students from the first through the fourth classes of a medical school of clinical medicine at a university located in Inner Mongolia in China. Informed consent was obtained. A total of 411 students was utilized with cluster sampling (probability sampling). The following cases were excluded from our study:

Students from other colleges.

Students with any mental illness.

Students other than 1st, 2nd, 3rd, and 4th year.

Noncooperative students.

### Measurement tools

The study tool was the ILS, which was constructed using the learning styles model by Felder-Silverman. It included 44 questions, each with two choices corresponding to four dimensions [18]. Hosford CC and Felder, R.M. et al. have confirmed the excellent reliability and validity of the ILS [12, 19–23].

### Specific operation

The class was taken as a unit in the specific operation, and a paper and pen test method was utilized. All questionnaires were answered voluntarily and anonymized before they were collected. First, basic information about the subjects was collected. The instructions of the questionnaire were read under guidance. The subjects answered the questions without a time limit, and the main experimenter received the questionnaire after all answers were completed. A total of 423 questionnaires were distributed to clinical college students of Inner Mongolia Minzu University in a cluster sample. A total of 411 valid questionnaires were obtained with an effective rate of 97%. Students were requested to choose the choice that best described their LS.

### Data analysis

SPSS software (version 17.0) was adopted for the statistical analysis. The current research calculated descriptive statistics. Furthermore, the chi-square indicated the importance of referential assessment at a 5% significance level. The reliability indices were obtained from answers to the ILS, and Cronbach’s α was used to evaluate the reliability of the internal consistency (IC) [22]. Exploratory factor analysis was utilized to evaluate the ILS scales’ construct validity [23].

## Results

### Demographic characteristics

A total of 411 clinical MSs completed the survey. Table 1 shows the participants’ demographic characteristics.

**Table 1:** Demographic Characteristics of Surveyed Students (N = 411)

Characteristic	No. (%)
Age (in years)	
Mean ± SD	21 ± 1.5
Gender	
Male	161 (39.17)
Female	250 (60.83)
Class	
Freshman	148 (36.01)
Sophomore	83 (20.19)
Junior	77 (18.73)
Senior	103 (25.06)

### Reliability and validity of ILS scores

Internal consistency reliability is attributed to the homogeneity of items considered for measuring similar quantities (e.g., the active/reflective priority); thus, the correlation of the responses to the items was obtained. Cronbach’s coefficient alpha, a mean of each feasible split pair correlation, is a standard measure of reliability [10]. The reliability coefficients in the present research for active-reflective, intuitive-sensitive, visual-verbal, and sequential-global were 0.52, 0.53, 0.58, and 0.50, respectively. Each alpha indicated an allowable (*>* 0.5) IC level for a priority metric [12, 19].

The current work generally employed investigative parameters to verify and evaluate the scale’s framework validity. Factor analysis was conducted to detect clusters of items for answers with the same variation pattern: “Each such cluster, or factor, is denoted by a group of variables whose members correlate more highly among themselves than they do with variables not included in the cluster” [24]. Solutions from four parameters were considered because the ILS relies on four LS scales. The convergence of rotations was reached after six iterations, and the four derived factors included 24.66% of the whole variance in item responses. A survey of the items in each factor is described in Table 2. The number of dimension items related to visual/verbal and active/reflective was eight and nine, respectively, and their scores greater than 0.2 were located in the first and the fourth components. The number of dimensions related to sensing/intuitive and sequential/global was eight and seven, respectively, and their scores greater than 0.2 were located in the second and the third components (although four items, 10, 38, 25, and 40, were not strongly located at that parameter). Table 2 presents items without primary weights for that parameter in boldface.

The response analysis supported the ILS’s desired framework and intermediate reliability.

**Table 2:** Factors in the four-factor solution for the ILS

Component	Items											Scale
1	19	27	23	31	3	11	7	35	**39**	**15**	**43**	Visual/Verbal
2	34	6	22	2	30	10	38	14	**18**	**26**	**42**	Sensing/Intuitive
3	28	44	32	4	36	20	40	**12**	**16**	**24**	**8**	Sequential/Global
4	41	21	1	29	37	13	9	17	25	**5**	**33**	Active/Reflective

### Student learning style distribution

**Fig. 1:**
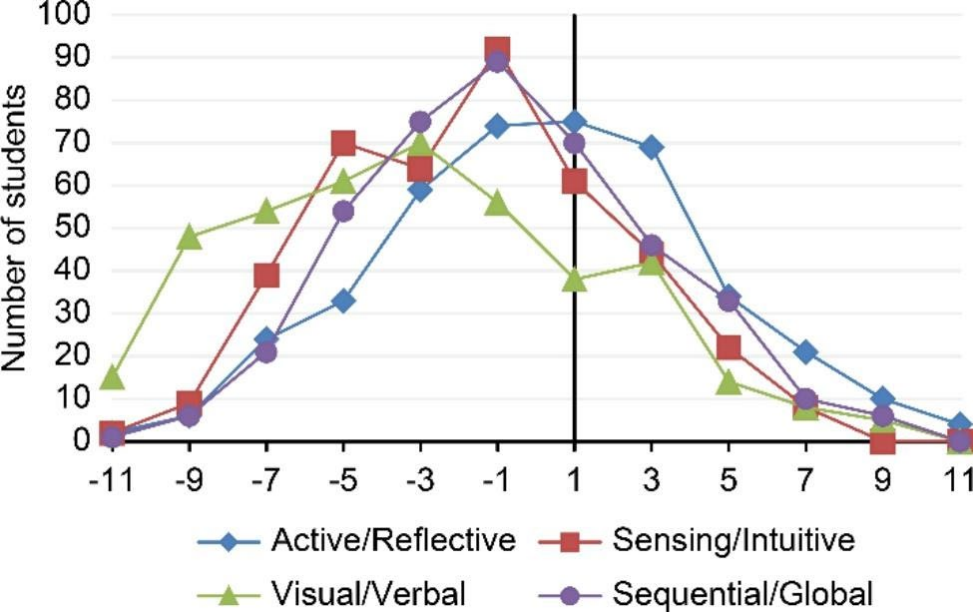
Distribution of all four dimensions of the students’ learning styles

Both Table 3; Fig. 1 show that students of medicine preferred visual, sensing, and sequential LSs. Their learning priority for all learning styles was determined by using a scale between 1 and 11, as described previously. The priorities of all students are presented in Fig. 1. Students’ sequential, sensing, and visual characteristics can be observed, and further balanced characteristics for the active/reflective dimension exist. Among the entire group, the main priority was visual.

**Table 3:** Overall Learning Style Results (N = 411)

Dimension N (%)	Number of Students
Active versus Reflective	
Active	198 (48.18)
Reflective	213 (51.82)
p value	> 0.05
Sensing versus Intuitive	
Sensing	276 (67.15)
Intuitive	135 (32.85)
p value	< 0.01
Visual versus Verbal	
Visual	304 (73.97)
Verbal	107 (26.03)
p value	< 0.01
Sequential versus Global	
Sequential	246 (59.85)
Global	165 (40.15)
p value	< 0.01

### Learning style preference by various student groups

Statistical analysis was conducted to detect the difference between learning styles for various categories to which students were assigned to compare the LSs between males and females. The priorities of male and female students were approximately the same for the whole domain (Table 4).

**Table 4:** Learning Style Preferences by Gender

Dimension N (%)	Males	Females
Active versus Reflective		
Active	69 (42.9)	129 (51.6)
Reflective	92 (57.1)	121 (48.4)
p value	> 0.05	
Sensing versus Intuitive		
Sensing	103 (64)	173 (69.2)
Intuitive	58 (36)	77 (30.8)
p value	> 0.05	
Visual versus Verbal		
Visual	111 (68.9)	193 (77.2)
Verbal	50 (31.1)	57 (22.8)
p value	> 0.05	
Sequential versus Global		
Sequential	88 (54.7)	158 (63.2)
Global	73 (45.3)	92 (36.8)
p value	> 0.05	

**Fig. 2:**
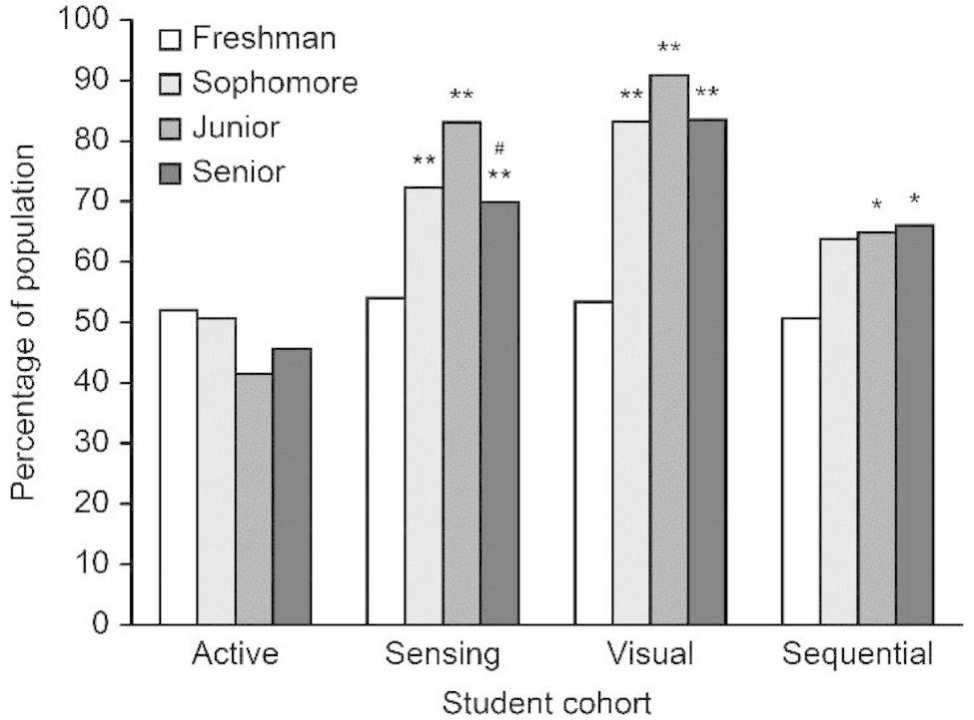
Learning style preference by grade Comparison between juniors and seniors, # *p* < 0.05 Comparison between freshmen and sophomores, juniors, seniors, ** *p* < 0.01 * *p* < 0.05

LS priority can be described by grade. The learning styles of various groups were different. For example, a considerable (p values of 0.01 and 0.05) distinction in percentage was observed between freshmen and students who were sophomores, juniors, and seniors with the sensor/visual/sequential LS priorities presented in Fig. 2. These data indicate that it is likely that a variety of learning styles are present in a given group of clinical medical students and that some variations in LS preferences can exist within groups of students of the same major at the same university.

**Fig. 3:**
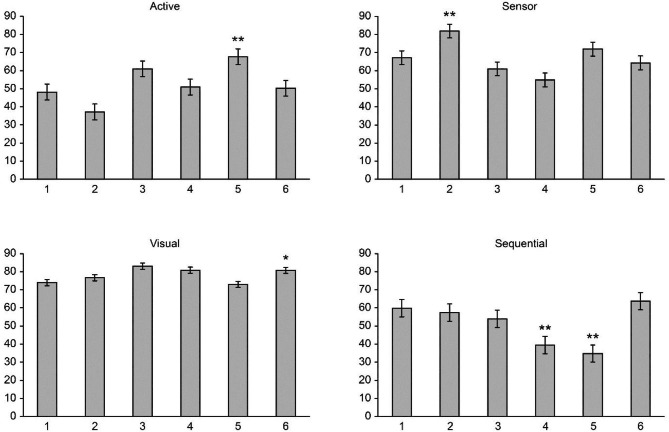
Preferred learning styles of Inner Mongolia Minzu University students compared to other medical students. 1 Inner Mongolia Minzu University; 2 Korea University; 3 Utrecht University; 4 Nazarbayev University; 5 Taibah University; 6 King Edward Medical University. ** *p* < 0.01, * *p* < 0.05

Data acquired from other published works (Fig. 3) have indicated that medical students have active, visual, sensing, and sequential LS priorities (except Nazarbayev and Taibah universities) [10, 17, 25, 26 and 27]. Our clinical medical students’ learning styles were basically the same as those of the other medical students, but the active/reflective style was more balanced (Fig. 3).

## Discussion

Felder and Silverman’s (1988) Index of Learning Styles, which originated in the engineering sciences, is defined as the characteristic preferences and strengths in the ways individuals take in and process information [1]. The ILS has been widely used to classify student learning styles in various majors [10, 28, 31].

The reliability and validity of ILS scores were analysed. Cronbach’s alpha is a popular metric for determining a questionnaire’s internal consistency for scales. A higher value of this measure indicates more reliability of the produced scale. Tuckman [29] indicated that alpha test reliability should exceed 0.75 and 0.5 for achievement and attitude tests, respectively. Felder and Spurlin [19] considered 0.5 the acceptable criterion for the ILS. The alpha values for all four scales of the ILS met this criterion, and 24.66% of the whole variability included the four components derived in the factor analysis. The above outcome is the same as that reported by Zywno and Waalen [21], including 28.9% of ILS variability by deriving five factors. Charles C. Hosford [12] indicated that the four derived components represented 27.5% of the whole variability in item responses. Approximately 60 − 80% of the items in the four dimensions were loaded onto the same factor analysis component. Response analysis supported the ILS’s desired framework and appropriate reliability.

According to the outcomes, students’ LS priorities were perceptual, sequential, and visual. Accordingly, as discussed in the literature, students majoring in the medical field prefer to master knowledge via demonstrations, photos, practice and familiarity with facts, diagrams, and algorithms sequentially and linearly [10, 26, 27]. Nevertheless, according to the analyses, no considerably significant differences were observed between priorities when continual active-reflective learning was a concern.

The impact of gender on the LSs of MSs should be analysed. For example, Daniel Hernández-Torrano indicated that male MSs favour visual learning over verbal learning when compared to female MSs. Compared with men, women prefer sequential learning to global learning [10]. Hosford & Sides [12] indicated that more female MSs prefer perceptual learning, while male MSs often prefer visual learning. In addition, the performed studies indicated that women prefer an active learning style, such as practice, doing, or attempting, as an alternative to a reflective style, such as thinking about problems [17]. In contrast, specific works have challenged the gender role in students’ LS priorities. This was also detected for the preference of reflective learning versus active learning for both females and males, and the difference between male and female students was not substantial [10]. Alghasham AA indicated no observable evidence between female and male medical students in learning style or learning preference using the ILS instrument [13]. The current research demonstrated that although the number of female students who preferred active and sensing LSs was greater than that of male students, the difference was insignificant.

Previous works have indicated that the dominant LSs for medical students were visual and sensing [10, 12–17, 25–27]. According to our outcomes, the dominant LSs for Chinese medical students were perceptual and visual, in line with the previously obtained outcomes. In addition, although some works have reflected medical students’ positive learning style priorities [17, 26], others have indicated that reflective or active information processing is an insignificant priority [12]. The obtained outcomes reflect more balance in the dimension of active/reflective learning and the dominance of the sequential learning style, which is compatible with the LS of King Edward Medical University MSs.

Indicating the LS priorities of students helps students employ the mentioned knowledge to improve the efficiency of their learning practices. According to a longitudinal analysis of courses related to chemical engineering, compared to courses related to control, courses established particularly to accommodate various student LSs can improve students’ trust in academic preparation [14] and overall academic efficiency [15], even in courses taught by other faculty via “traditional” approaches [14], thereby improving student preservation [27] and the number of individuals who graduate. Thus, teachers should employ teaching approaches that have maximum compatibility with this type of student. Ross [30] stated that the compatibility of teachers’ teaching approaches and training materials with students’ preferred learning approaches facilitates learning activities, promotes learning performance, and accelerates learning. In “Chemical Methodology” teaching, Felder & Silverman’s LS was employed to analyse the LSs of students majoring in chemistry. They were categorized into various learning classes based on their LS differences and established various learning approaches and teaching actions by Liu et al. [31]. This innovative learning approach can enhance students’ compatibility and develop their corresponding learning approaches through teaching practice.

The current study verifies the existence of a spectrum of learning styles among clinical medical students and extends an incentive for teachers to understand how their teaching styles can incorporate multiple styles of learning.

### Limitations and future research

Since this is the first study to verify the LS of medical students from China, it has various restrictions. First, since the samples were acquired from a single university in China, the generalization of the results may be restricted. Second, this study concentrated only on undergraduate students without considering postgraduate students, who may have different beliefs. Third, learning styles can be developed and changed through learning experiences [18], and the current work is a one-shot study that cannot describe the dynamic behaviour of LSs.

The following can be considered future research avenues. First, similar research should be performed considering higher education institutions in China. Second, a longitudinal analysis should be performed while considering the dynamic behaviour of LSs. Additionally, research associating LSs and annual test results for Inner Mongolia Minzu University MSs would be of interest.

## Conclusions

In summary, our outcomes indicated that the preferred LSs of students majoring in clinical medicine at Inner Mongolia Minzu University were perceptual, visual, and sequential. This finding is similar to the LS characteristics of MSs in other universities. This study can provide valuable information to construct efficient learning actions at Inner Mongolia Minzu University. This study provides a firm foundation on which to base further studies of this cohort of students as they progress through medical education.

## Data Availability

The data generated and analysed in this study are included in this published article. The raw dataset analysed is available from the corresponding author upon reasonable request.

## Abbreviations

ILS: Index of Learning Styles
LS: learning style
MS: medical student
